# Facile and reproducible method of stabilizing $\hbox {Bi}_2\hbox {O}_3$ phases confined in nanocrystallites embedded in amorphous matrix

**DOI:** 10.1038/s41598-021-98435-5

**Published:** 2021-09-27

**Authors:** Tomasz K. Pietrzak, Agata Jarocka, Cezariusz Jastrzębski, Tomasz Płociński, Marek Wasiucionek, Jerzy E. Garbarczyk

**Affiliations:** 1 Faculty of Physics, Warsaw University of Technology, Koszykowa 75, 00-662 Warsaw, Poland; 2 Faculty of Materials Science and Engineering, Warsaw University of Technology, Wołoska 141, 02-507 Warsaw, Poland

**Keywords:** Applied physics, Chemical physics, Condensed-matter physics

## Abstract

Bismuth sesquioxide ($\hbox {Bi}_2\hbox {O}_3$) draws much attention due to wide variety of phases in which it exists depending on the temperature. Among them, $\delta$ phase is specially interesting because of its high oxide ion conductivity and prospects of applications as an electrolyte in fuel cells. Unfortunately, it is stable only in a narrow temperature range ca. 730–830 $^{\circ }$C. Our group has developed a facile and reproducible two-stage method of stabilizing $\hbox {Bi}_2\hbox {O}_3$ crystalline phases confined in nanocrystallites embedded in amorphous matrix. In the first stage, glassy materials were obtained by a routine melt-quenching method: pure $\hbox {Bi}_2\hbox {O}_3$ powders were melted in porcelain crucibles and fast-cooled down to room temperature. In the second step, the materials were appropriately heat-treated to induce formation of crystallites of $\beta$, $\delta$ or $\gamma$ $\hbox {Bi}_2\hbox {O}_3$ phases confined in a glassy matrix, depending on the process conditions. It was found out that the vitrification of the initial $\hbox {Bi}_2\hbox {O}_3$ and the subsequent nanocrystallization were unexpectedly possible due to the presence of some Al, and Si impurities from the crucibles. Systematic DTA, XRD, optical, Raman and SEM/EDS studies were carried out to investigate the influence of the syntheses processes and allowed us to determine conditions under which the particular phases appear and remain stable down to room temperature.

## Introduction

Bismuth sesquioxide ($\hbox {Bi}_2\hbox {O}_3$) draws much attention due to a wide variety of phases which it adopts depending on the temperature. Among them, the $\delta$ phase exhibits a record high conductivity reaching 1 $\hbox {Scm}^{-1}$ at 750 $^\circ$C. For this reason, it has been considered a leading candidate for a ceramic electrolyte in in intermediate temperature solid oxide fuel cells (IT-SOFCs)^1,2^. Unfortunately, this phase is only stable in a narrow temperature range ca. 730–830 $^{\circ }$C^3^. Below 730 $^\circ$C it transforms to metastable and poorly conducting $\beta$- or $\gamma$-phases^3^. The $\beta$ phase has also drawn much scientific interest due to its valuable optical properties and prospective applications in photocatalysis. However, the main phase stable at room temperature is the monoclinic $\alpha$ phase. Many attempts have been made to retain the defective fluorite structure of $\delta$-$\hbox {Bi}_2\hbox {O}_3$ to temperatures below 730 °C. Most of them have involved solid solutions of $\hbox {Bi}_2\hbox {O}_3$ with substantial fractions of selected oxides (e.g.^4,5^). In fact, such polycrystalline materials presented the fluorite-like ordering even at room temperature, but their conductivity was disappointingly much lower than expected from a $\delta$-$\hbox {Bi}_2\hbox {O}_3$—like structure^4,5^.

In the last decades, several articles have reported that it is possible to extend downwards the stability range of certain simple compounds high-temperature crystalline phases using a very different approach. One of the first articles on such an effect was published in 1991 by Tatsumisago et al. in Nature^6^. They reported that a very rapid cooling ($> 10^5$ K/s) of silver borate glasses very rich in AgI led to the formation of composite glass-ceramic solids with nanosized crystallites of $\alpha$-AgI evenly dispersed in the glassy matrix. The most surprising was the presence of the $\alpha$-phase at room temperature, knowing that in a bulk form this phase is stable only above 147 $^{\circ }$C. Funke et al. stated that this phenomenon was due to the confinement effects of nanocrystallites embedded in a glassy matrix^7^.

A strikingly similar situation was observed by our group in the case of bismuthate glasses^8^. It was observed that the annealing of (nearly) pure $\hbox {Bi}_2\hbox {O}_3$ glasses at a temperature slightly higher than their glass transition temperature $T_g$ led to the formation of nanosized crystallites of a high-temperature $\delta$-$\hbox {Bi}_2\hbox {O}_3$ phase confined inside the glassy matrix. The resulting materials, including $\delta$-$\hbox {Bi}_2\hbox {O}_3$ crystalline grains, were stable at room temperature for long periods of time (even over a year), despite the fact that the bulk $\delta$-$\hbox {Bi}_2\hbox {O}_3$ is stable in the 730–830 $^{\circ }$C range only^3^. Hence, an idea of a facile way to inexpensive and facile syntheses of bulk amounts of nanomaterials of nanocrystallites of $\delta$-$\hbox {Bi}_2\hbox {O}_3$ embedded in a residual glassy matrix arises. It consists of two stages only: (i) melting pure $\hbox {Bi}_2\hbox {O}_3$ in silica/alumina crucibles followed by their rapid quenching; (ii) heating the samples to ca 630 $^{\circ }$C. Later, the same approach was used by Margha et al.^9^, who succesfully synthesized glass ceramics of more complex $\hbox {Bi}_2\hbox {O}_3$–$\hbox {BiFeO}_3$ system, containing the $\beta$ and $\gamma$ phases of $\hbox {Bi}_2\hbox {O}_3$.

In both situations ($\alpha$-AgI and $\delta$-$\hbox {Bi}_2\hbox {O}_3$) there is no consistent and well-evidenced explanation of the mechanisms responsible for the stabilization of high temperature phases down to room temperature. Our hypothesis is that this phenomenon is due to the confinement of nanocrystallites of high-temperature phases in glassy matrices. This confinement does induce non-negligible interactions between the interiors of these crystallites, their surfaces, and the embedding glassy phase. These factors may cause a shift of local conditions acting on the nanosized crystallites of high-temperature phases, compared to those of the bulk phase. This shift may favor the stability of the high-temperature phase as long as it remains confined.

While the final explanation of the aforementioned mechanism still requires far fetched studies, in this work we attempted to identify experimental parameters that have a significant influence on phase stabilization in our bulk glass-nanoceramics. In comparison to the work reported in Ref.^8^, here we synthesized several samples with different melting times and extensively studied them by a range of methods, probing structure, thermal properties, microstructure, optical transitions and local ordering and dynamics: X-ray diffractometry (XRD), differential thermal analysis (DTA), scanning electron microscopy with energy dispersive spectroscopy (SEM/EDS), Raman spectroscopy and optical absorption measurements.

XRD studies allowed us to confirm the amorphousness of as-synthesized samples and identify the phases that appeared after heat treatment. The temperatures were correlated with the results of DTA measurements. SEM was used to investigate the microstructure of the samples and the elemental composition was determined with EDS. Micro-Raman spectroscopy can provide important information on the local structure in vitreous and ceramic materials. In this work, Raman spectroscopy was applied to probe the homogeneity of the samples and to detect the precipitation of other phases in the structure of $\hbox {Bi}_2\hbox {O}_3$. Absorption spectroscopy provided information about the absorption edge in the material and hence helped to determine values of the optical gap.

**Figure 1:**
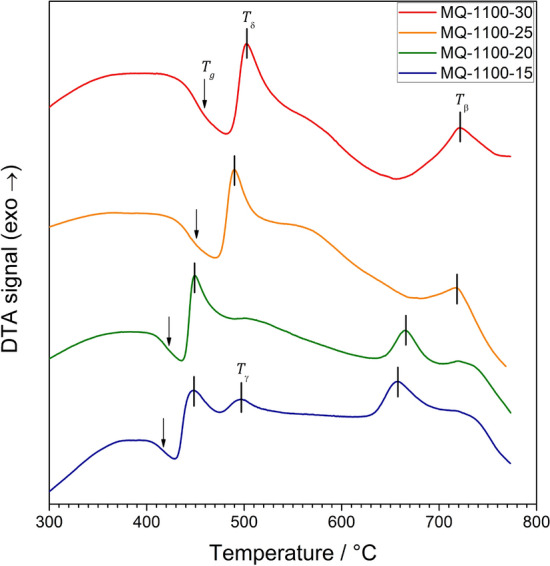
DTA traces of glassy samples synthesized under different conditions (melting time from 15 to 30 min at 1100 $^{\circ }$C). Glass transition and crystallization thermal events are marked. The phases were ascribed to crystallization processes according to XRD results.

## Results

### Differential thermal analyses (DTA)

DTA traces of as-synthesized glasses are shown in Fig. 1. All glasses had been synthesized at 1100 $^{\circ }$C. The batches differed in the melting time, which varied from 15 to 30 min (Table 1). Their DTA traces are typical for glassy materials. A step-like endothermic shift of the baseline, characteristic for glass transition, is followed by several exothermic peaks related to crystallization phenomena. One can see that the temperatures of these thermal events depend on the sample synthesis conditions. In general, the longer melting time, the higher these temperatures are. The temperature of glass transition ($T_g$) varies between 418 and 450 $^{\circ }$C. The maximum of the main crystallization peak ($T_\delta$) can be found in the range of 442–502 $^{\circ }$C. All the temperatures are listed in the Table 2.

**Table 1:** List of the synthesized glassy samples with the process parameters (melting temperature and time) and the relative atomic content of Al and Si dopants.

Sample ID	Melt. temp. (°C)	Melt. time	Al:Bi	Si:Bi
MQ-1100-15	1100	15	0.345	0.288
MQ-1100-20	1100	20	0.372	0.323
MQ-1100-25	1100	25	0.338	0.353
MQ-1100-30	1100	30	0.352	0.398

**Table 2:** Temperatures of glass transition $T_g$ and crystallizations ($T_\delta$, $T_\gamma$, $T_\beta$) assigned to $\beta$, $\delta$ and $\gamma$-$\hbox {Bi}_2\hbox {O}_3$ phases.

Sample ID	$T_g$	$T_\delta$	$T_\gamma$	$T_\beta$
MQ-1100-15	418	443	497	656
MQ-1100-20	419	448	–	665
MQ-1100-25	443	489	–	719
MQ-1100-30	450	502	–	719

All values are given in $^{\circ }$C.

### X-ray diffractometry (XRD)

**Figure 2:**
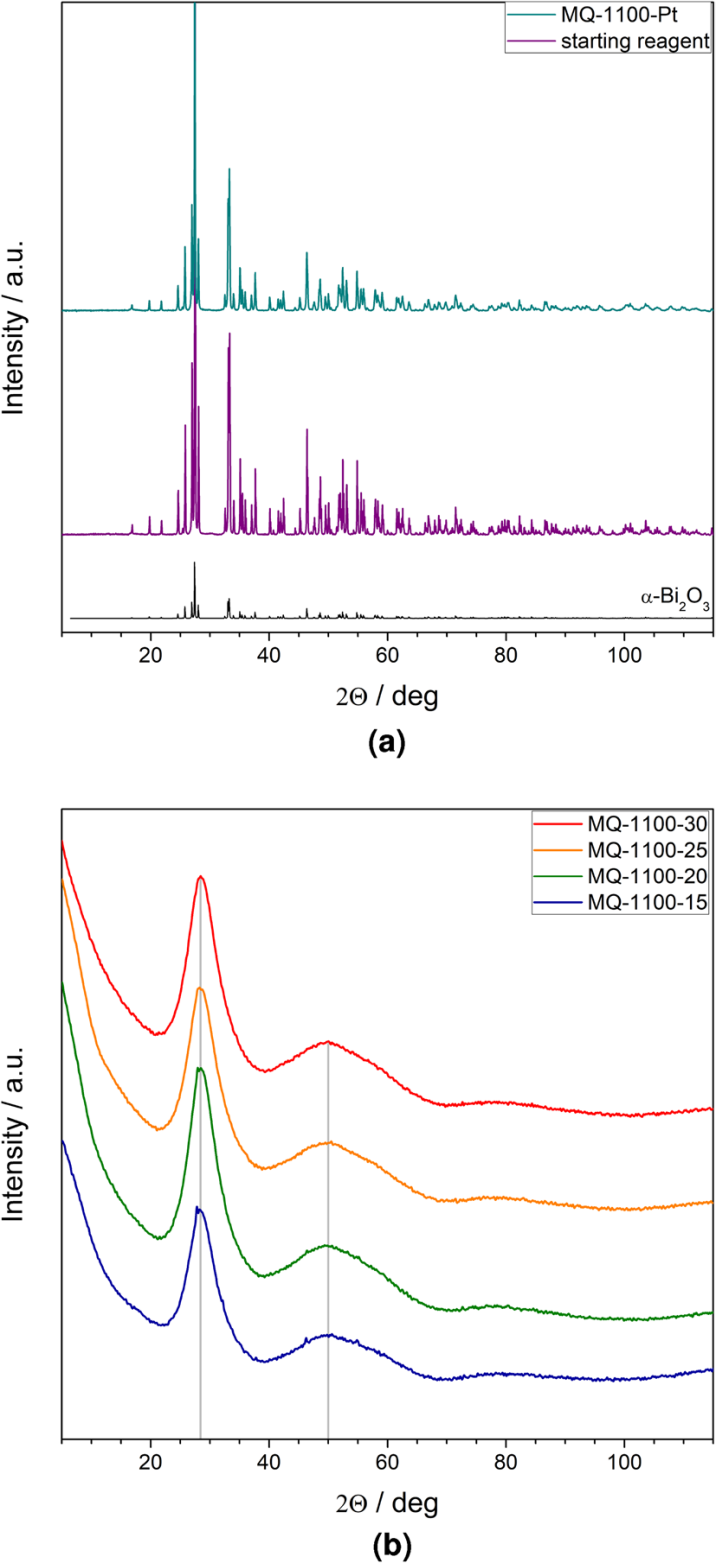
XRD patterns of: (**a**) starting $\hbox {Bi}_2\hbox {O}_3$ powder (reagent) and samples melt-quenched in a platinum crucible; $\alpha$-$\hbox {Bi}_2\hbox {O}_3$ reference pattern is given in the bottom; (**b**) glassy samples processed in porcelain crucibles;distinctive amorphous *halos* are observed at $2 \Theta = 28.4 ^{\circ }$ and $2 \Theta = 50.1 ^{\circ }$.

The diffraction patterns of the commercially purchased $\hbox {Bi}_2\hbox {O}_3$ reagent, which was used for all syntheses reported in this work, and the sample melted in a platinum crucible and subsequently rapidly cooled to room temperature are shown in Fig. 2a. It came not as a surprise that the starting reagent adopted the thermally stable $\alpha$-$\hbox {Bi}_2\hbox {O}_3$ phase. The collected diffractogram was in good agreement with ICDD reference pattern no. 04-007-1342 (shown in the figure for comparison). The sample melted in a platinum crucible and subsequently quenched exhibited the same structure, i.e. $\alpha$-$\hbox {Bi}_2\hbox {O}_3$.

The patterns of the samples synthesized in porcelain crucibles are shown in Fig. 2b. Their shape is typical for amorphous materials. No distinct Bragg reflexes are present. On the contrary, two very broad and intensive halos centered at $2 \Theta = 28.4 ^{\circ }$ and $2 \Theta = 50.1 ^{\circ }$, respectively, are visible.

**Figure 3:**
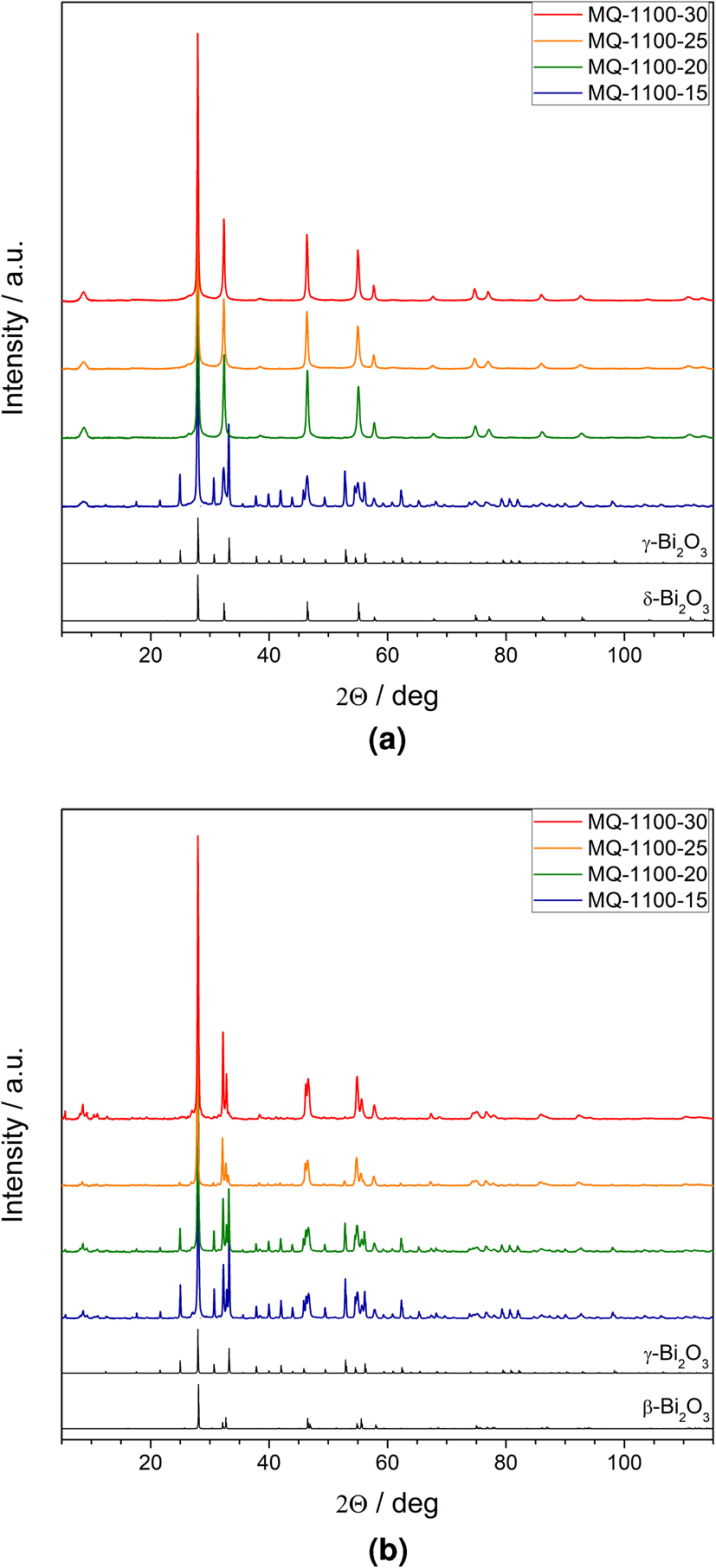
Room temperature XRD patterns of samples after their heat-treatment at 630 $^{\circ }$ C (top) and 730 $^{\circ }$ C (bottom). $\beta$, $\delta$ and $\gamma$-$\hbox {Bi}_2\hbox {O}_3$ reference patterns (ICDD cards no. 04-008-4464, 04-015-0028 and 04-007-2465, respectively) are given in the bottom.

Room temperature XRD patterns of the samples that were heat-treated at $T_{max} =$ 630 and 730 $^{\circ }$C are presented in Fig. 3a,b. One can clearly see that both glass synthesis conditions and temperatures $T_{max}$ have impact on the crystalline phases emerging due to the heat-treatment at $T_{max}$. Three crystalline phases were identified: $\beta$ (ICDD card no. 04-008-4464, tetragonal, space group P-421c), $\delta$ (ICDD card no. 04-015-0028, fcc, space group Pn-3m) and $\gamma$ (ICDD card no. 04-007-2465, cubic, space group I23). The following samples contained pure $\delta$-$\hbox {Bi}_2\hbox {O}_3$ phase: MQ-1100-20, MQ-1100-25 and MQ-1100-30—after heated to 630 °C. Additionally, in some samples minor traces of hexagonal $\hbox {SiO}_2$ were found (ICDD card no. 01-075-3168). The phase composition of all studied samples is shown in Table 3.

**Table 3:** Phase composition (in vol percent) of the materials under study after their thermal treatment at $T_{max} =$ 630 and 730 $^{\circ }$ C.

Sample ID	$T_{max} = 630\,^{\circ}$ C	$T_{max} = 730\,^{\circ}$ C
MQ-1100-15	$\gamma$ (58 %) / $\delta$ (42 %)	$\gamma$ (56 %) / $\beta$ (46 %)
MQ-1100-20	$\delta$	$\gamma$ (49 %) / $\beta$ (51 %)
MQ-1100-25	$\delta$	$\beta$
MQ-1100-30	$\delta$	$\beta$

### Scanning electron microscopy (SEM) with energy dispersive X-ray spectroscopy (EDS)

**Figure 4:**
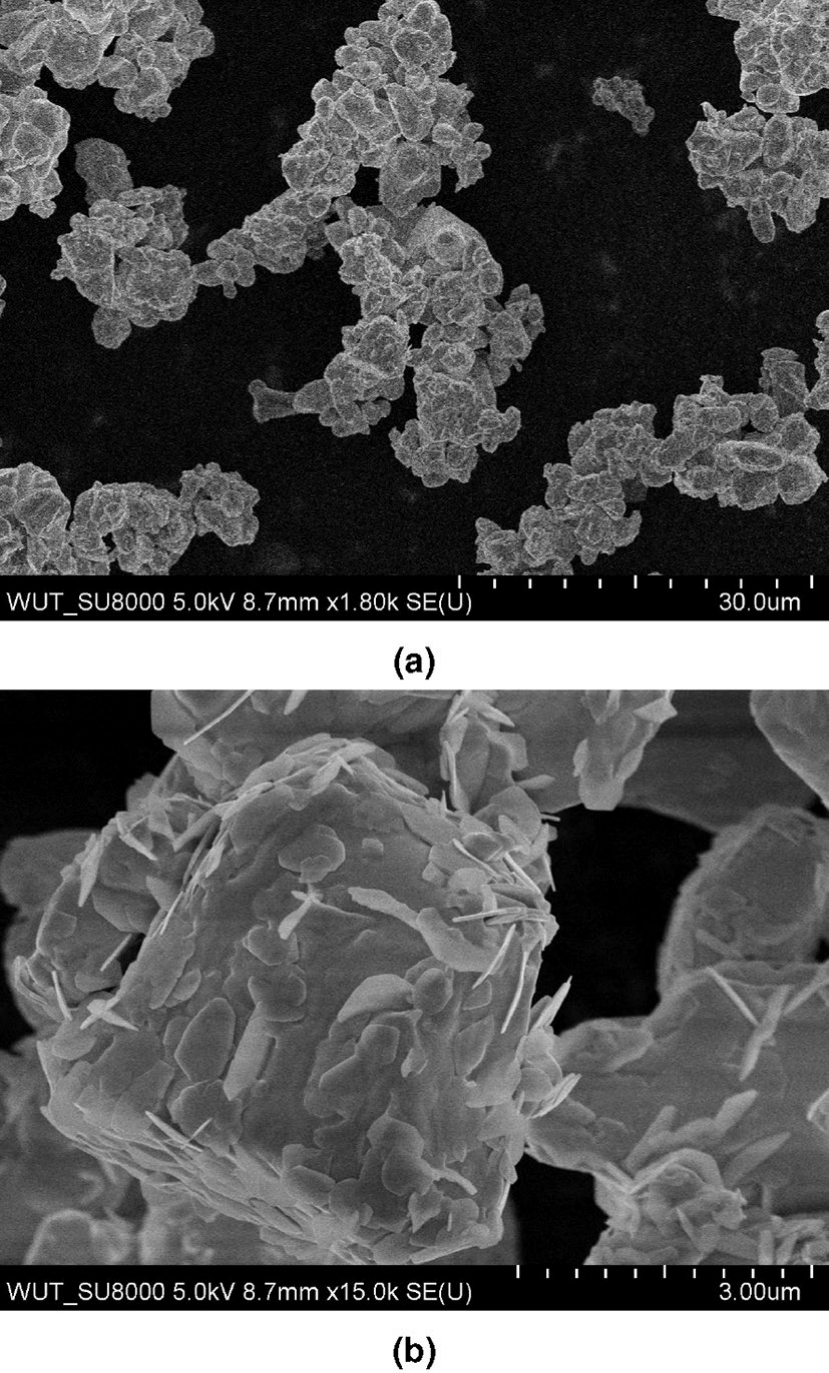
SEM images acquired at different magnifications of the starting $\hbox {Bi}_2\hbox {O}_3$ powder used for all syntheses in this work. Two different parts of the reagent with various magnifications represent typical shapes of grains.

EDS analysis showed no impurities in the starting $\hbox {Bi}_2\hbox {O}_3$ powder, confirming its high purity, declared by the seller. The material consisted of microscopic grains. Typical sizes varied between 1 and 5 µm. Nevertheless, considerably bigger particles were also observed (Fig. 4a,b).

The structure of the crucibles used for syntheses was very interesting. Their inner part was porous. In our case, it means that it could be easily soaked with melted $\hbox {Bi}_2\hbox {O}_3$. The external part of these crucibles was glazed, which ensured much lower porosity. Nevertheless, the elemental compositions (investigated with EDS) of both the interior and exterior parts of the crucible were the same: O—73%, Si—19.5%, Al—5.5%, Mg—1.1%, Na—0.9%. The relative uncertainties of this method were estimated as 3%.

**Figure 5:**
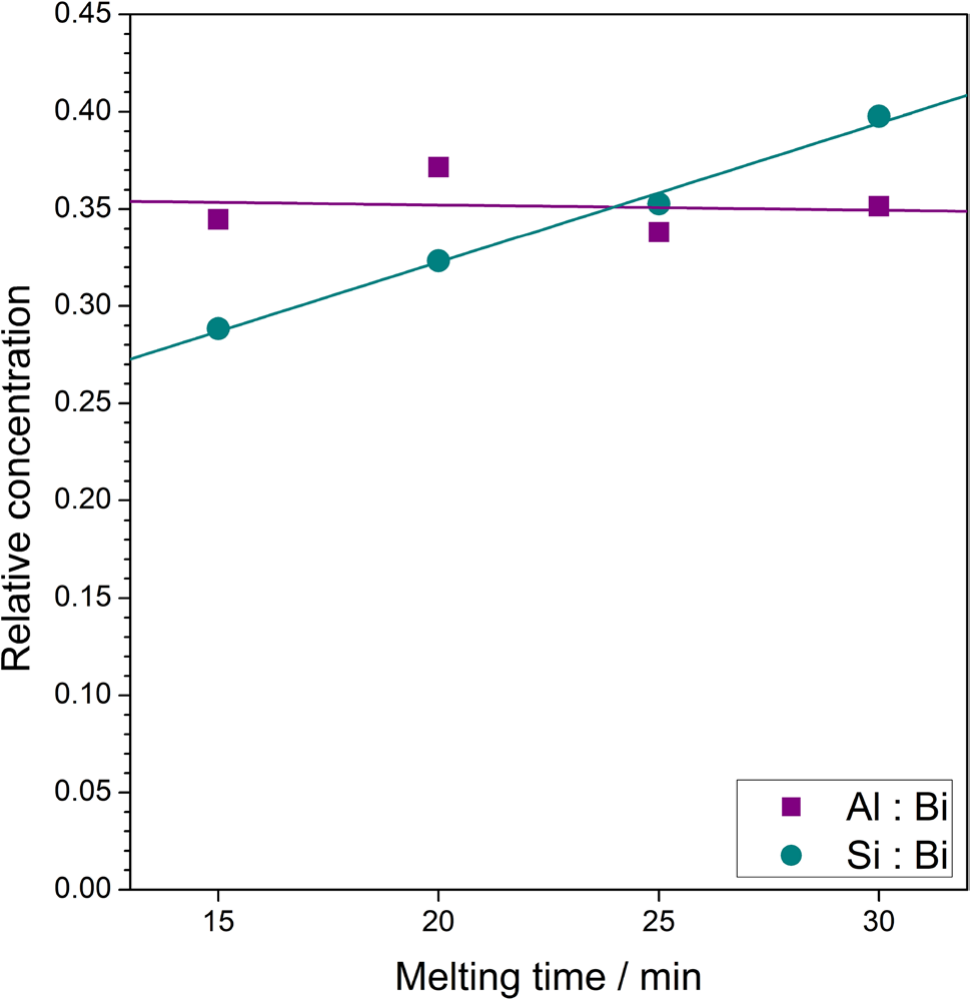
Relative concentrations of Si and Al dopants in the function of melting time during syntheses. Values are given as atomic Si:Bi and Al:Bi ratios and were determined from EDS analyses. Al content remains approximately constant regardless the melting time, while Si dopant linearly increases.

Being aware of the effect of the crucibles on the glasses prepared in these crucibles, we have determined (by EDS) the elemental composition of all glassy samples under study. Besides the lines originating from Bi and O, the fingerprints of Si and Al were spotted in all the samples. Therefore, quantitative analyses were performed and the atomic contents of Bi, O, Al and Si were determined. The relative contents Al:Bi and Si:Al are shown in Fig. 5. One can see that the content of Al remains approximately constant, whereas the content of Si increases with the increasing time of melting during synthesis. While at this stage it is too early to propose any model describing this phenomenon, the experimental linear correlation between Si content in the batch and the melting time is quite obvious.

**Figure 6:**
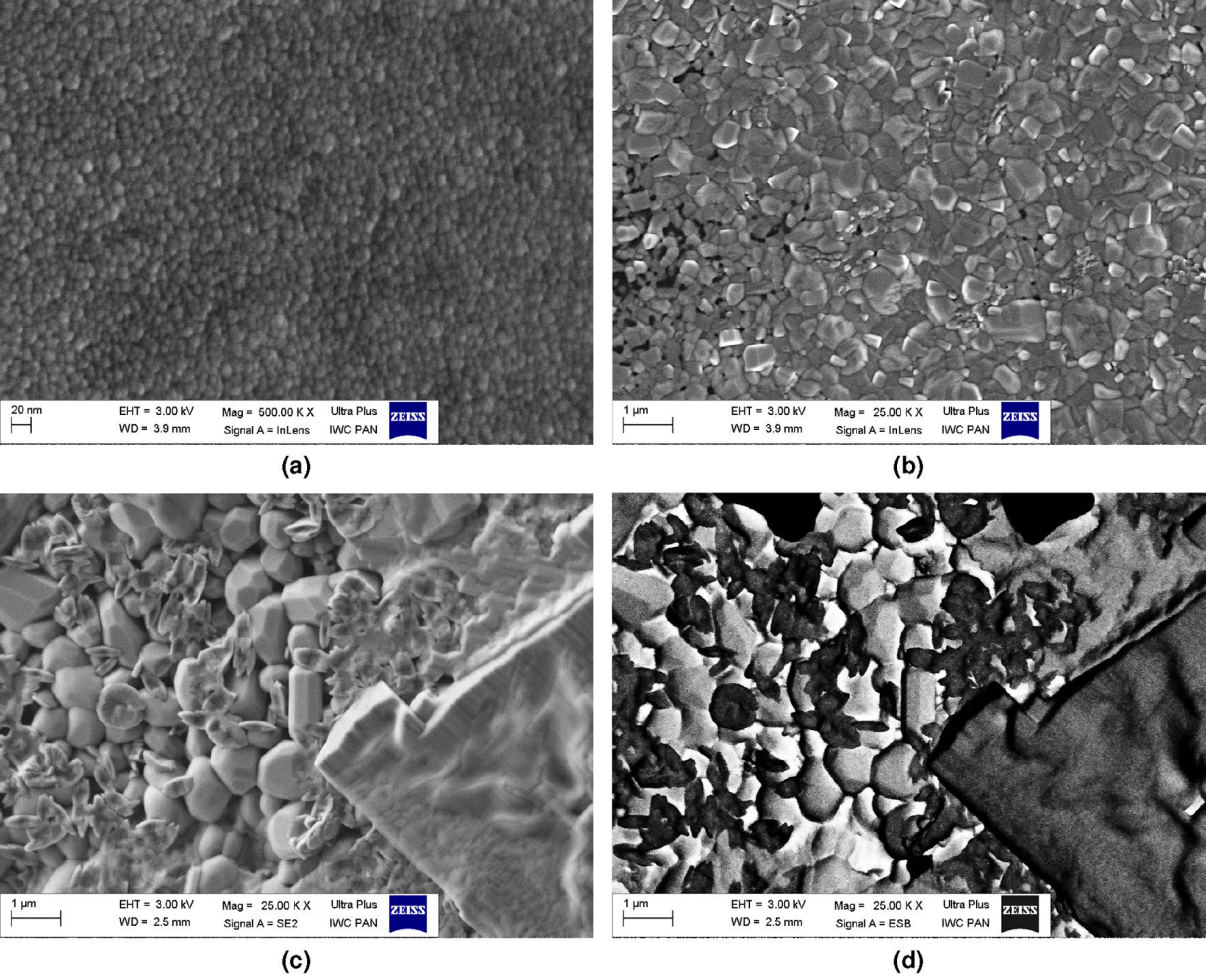
Representative SEM images of three phases—(**a**) $\delta$ – MQ-1100-30 heated up to 630 $^{\circ }$C; (**b**) $\beta$—MQ-1100-30 heated up to 730 $^{\circ }$C; (**c,d**) $\gamma$ – MQ-1100-15 heated up to 730 $^{\circ }$C—stabilized in glass-ceramics. One can see that average size of grains increases significantly from ca. 10–20 nm (for $\delta$ phase) to sub-micron (for $\gamma$ phase). Differences in local compositions were pronounced only for the samples with the $\gamma$ phase, as evidenced in electron backscattered mode (**d**).

SEM images of selected representative glasses after their thermal treatment revealed considerably different microstructure (Figs. 6a–c). The samples containing the $\delta$ phase only consisted of small (ca. 10–20 nm) nanocrystallites uniformly distributed in the bulk sample. Noticeably larger gains (i.e. sub-micron) were observed in the samples, where the pure $\beta$ phase was identified. On the contrary to the former samples, the sizes of grains of the $\gamma$ phase in the latter sample exceeded 1 µm. A pronounced inhomogeneity was also observed in such cases (Fig. 6d).

### Raman spectroscopy

**Figure 7:**
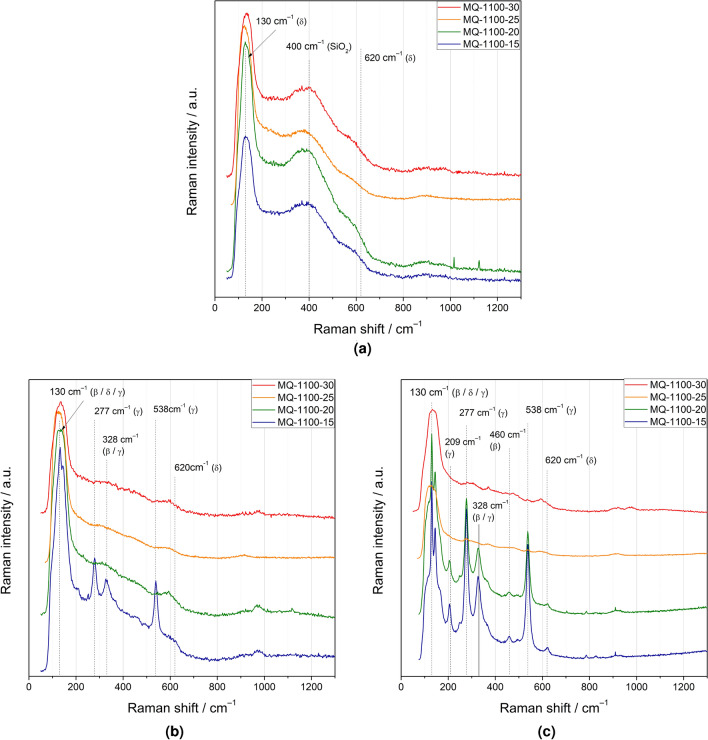
Room temperature Raman spectra of as-synthesized glasses (**a**) and the samples after heat-treatment at 630 $^{\circ }$C (**b**) and 730 $^{\circ }$C (**c**). The peaks have been tentatively assigned to various crystalline phases of $\hbox {Bi}_2\hbox {O}_3$.

Room temperature Raman spectra of as-synthesized glasses and the samples after an appropriate heat-treatment are shown in Fig. 7. The spectra of the glassy samples contain several broad maxima: below 200 $\hbox {cm}^{-1}$, around 400 $\hbox {cm}^{-1}$ and 620 $\hbox {cm}^{-1}$. The spectra of crystallized samples are more complex. In general, the samples originating from batches: MQ-1100-15 after heat-treatment at 630 $^{\circ }$C, and from MQ-1100-15 and MQ-1100-20 treated at 730 $^{\circ }$C contain several sharp lines, centered at 277, 328 and 538 $\hbox {cm}^{-1}$ (Fig. 7b,c). With the appearance of additional peaks, we observe the splitting of a wide peak located at about 130 $\hbox {cm}^{-1}$ in the aforementioned samples. Crystallized samples originating from batches MQ-1100-20 and MQ-1100-25 and, especially, MQ-1100-30 exhibit either low intensity modes or no maxima at these Raman shifts. On the contrary, a broad maximum at 620 $\hbox {cm}^{-1}$ is in this case more pronounced. This peak is partially overlapped for as-synthesized glasses by a wide band at about 400 $\hbox {cm}^{-1}$ and only the right-side shoulder of this band at about 600 $\hbox {cm}^{-1}$ is visible (Fig. 7a).

### Optical characterization

**Figure 8:**
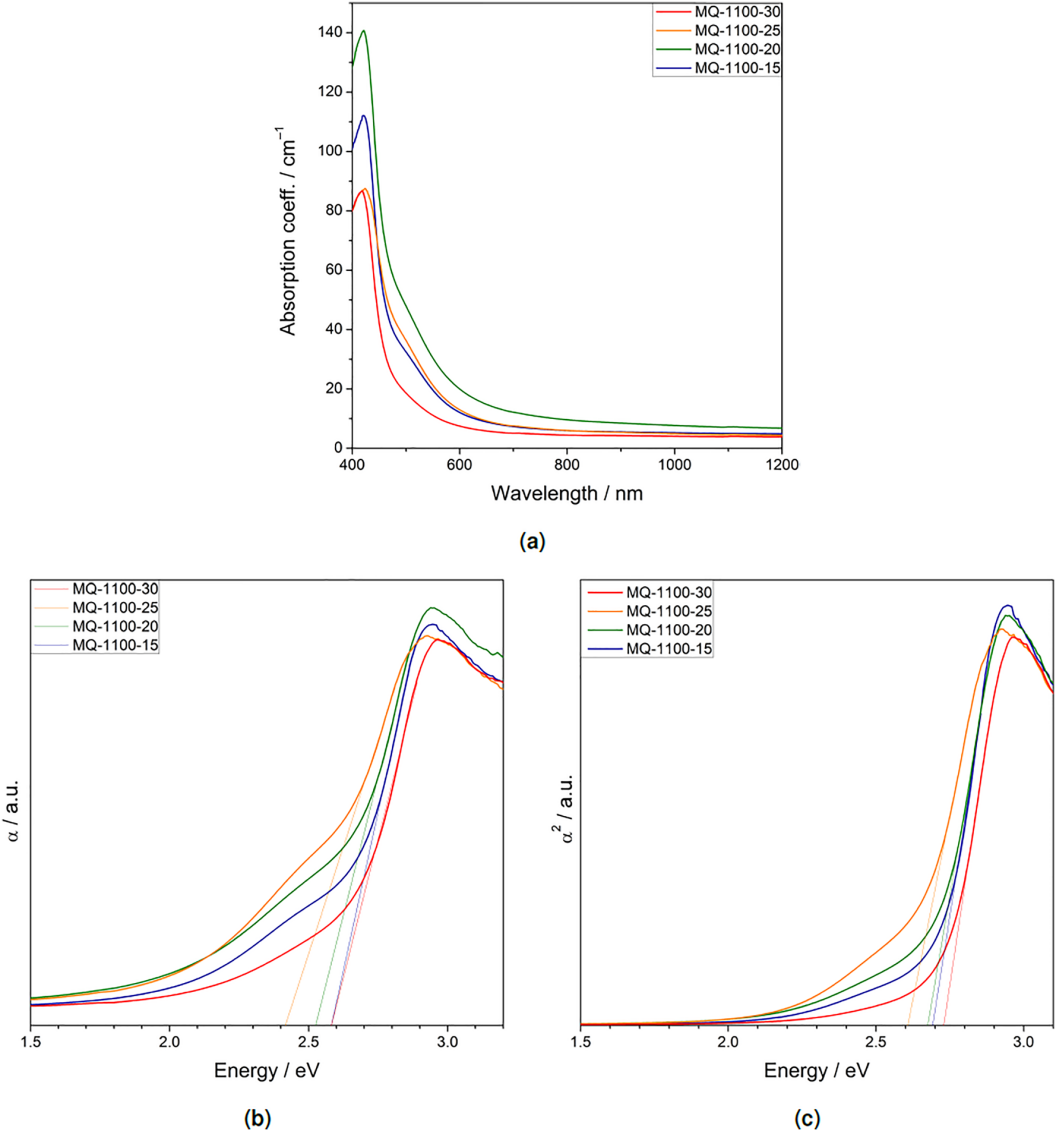
Absorption coefficient for synthesized glasses as a function of incident wavelength (**a**). Subsequently, the data were plotted in $\alpha$ vs. $h \nu$ (**b**) and $\alpha ^2$ vs. $h \nu$ (**c**) coordinates and normalized to determine the value of the optical gap.

In optical absorption measurements, the absorption edge for all studied glasses was within the visible range (Fig. 8a). Its shift towards lower wavelengths was observed with the increasing content of Si dopant (cf. Table 1). The first and second powers of the absorption coefficient $\alpha = \frac{1}{d} \ln \left( \frac{I_0}{I} \right)$ (where *d* is the thickness of the sample) were plotted versus $h \nu$ (Fig. 8b,c). Generally, for analysis and characterization of electronic absorption in solids within the UV–Vis–NIR, the most common is to represent the absorption coefficient as a function of energy or wavelength. Usually the dependency follows exponential relationship, where three main areas can be distinguished: (a) in higher energies—interband region (Tauc region), later on (b) Urbach edge region and in the lowest energies—charge transfer band defect states. Although, this dependence is much more defined for crystalline materials than for amorphous ones^10,11^.

Absorption edge of amorphous semiconductors enables to define an optical gap and can be described by a power law^11,12^. Mostly when it comes to glasses, one cannot determine if it is actual energy gap in states’ density. Therefore, it is more often considered as mobility gap ($E_0$) or just called optical gap or optical absorption edge^12,13^. The linear parts of the curves were extrapolated to $\alpha = 0$ and hence the values of optical gap $E_0$ for each sample were estimated. From the $\alpha (h \nu )$ plot, the results are as follows: 2.58, 2.52, 2.42 and 2.58 eV for samples MQ-1100-15, 20, 25, and 30, respectively. The $\alpha ^2 (h \nu )$ coordinates yielded slightly different values: 2.69, 2.68, 2.61, 2.73 eV, respectively. There is no monotonic dependence of the optical band gap on the composition of the glasses. Rather, we may assume that the values are scattered around 2.50 eV and 2.67 eV in the first (i.e. linear) and the second (i.e. $\alpha ^2 (h \nu )$) approach, respectively.

## Discussion and conclusions

In this paper, we focused on numerous technological aspects of the syntheses of $\hbox {Bi}_2\hbox {O}_3$ glasses and their influence on the phenomenon of stabilization of different phases embedded in a glassy matrix. It was shown that the attempts to obtain glassy $\hbox {Bi}_2\hbox {O}_3$ with melt-quenching technique using Pt crucibles failed. The material without impurities/dopants crystallized in a monoclinic $\alpha$ structure, which is the stable low temperature phase in polycrystalline bismuth oxide.

Use of porcelain crucibles, composed mainly of $\hbox {SiO}_2$ and $\hbox {Al}_2\hbox {O}_3$, resulted in significantly different outcomes. EDS experiments proved the solubility of these two oxides in melted bismuth oxide. The relative concentration of $\hbox {SiO}_2$ increased with the melting time, whereas the content of $\hbox {Al}_2\hbox {O}_3$ remained constant. The presence of these two glass formers / modificators made it possible to obtain glassy samples in $\hbox {Bi}_2\hbox {O}_3$–$\hbox {SiO}_2$–$\hbox {Al}_2\hbox {O}_3$ ternary system. The glasses exhibited differences in glass transition and crystallization temperatures. Similar behavior was observed previously for $\hbox {V}_2\hbox {O}_5$–$\hbox {P}_2\hbox {O}_5$ glasses synthesized with different time or temperatures^14^.

Very interesting results appeared when the glasses were heat-treated up to 630 and 730 $^{\circ }$C. The synthesis conditions had a considerable influence on the phases that appeared in the samples. In general, the desirable $\delta$ phase was stabilized down to room temperature in samples heated up to 630 $^{\circ }$C, whereas heating the samples to 730 $^{\circ }$C resulted in stabilization of the $\beta$ phase—another important for applications polymorph of $\hbox {Bi}_2\hbox {O}_3$. The batches with short melting time created advantageous conditions for facile crystallization of the second phase—gamma.

Raman studies are extremely useful for identifying small amounts of another crystalline or amorphous phase in a given sample. This is very important in the case of materials exhibiting polymorphism^15^. In the case of $\hbox {Bi}_2\hbox {O}_3$, the best known is its monoclinic $\alpha$ phase. The Raman spectrum of this phase contains many characteristic peaks^16^. In general, low-frequency peaks (i.e. below 200 $\hbox {cm}^{-1}$) are associated with the vibrations of Bi atoms, while those with frequencies above 200 $\hbox {cm}^{-1}$ are the result of vibrations of oxygen atoms with respect to the Bi atom causing Bi–O elongation or symmetric stretching of Bi–O–Bi bonds. In other phases of $\hbox {Bi}_2\hbox {O}_3$, also high-frequency vibrations (over 600 $\hbox {cm}^{-1}$) are observed.

Raman spectra of the $\hbox {Bi}_2\hbox {O}_3$ $\delta$-phase has only one Raman active phonon of $\hbox {T}_{{g2}}$ symmetry and thus one broad peak at 630 $\hbox {cm}^{-1}$ is reported^17–20^. The $\beta$-$\hbox {Bi}_2\hbox {O}_3$ phase exhibits Raman bands at 125, 314, 376, and 460 $\hbox {cm}^{-1}$^21^. The Raman peaks at 144, 278, 320, 384, 450 and 550 $\hbox {cm}^{-1}$ are attributed to $\gamma$-$\hbox {Bi}_2\hbox {O}_3$^22–24^. Moreover, in these $\hbox {Bi}_2\hbox {O}_3$ phases there are many other peaks of low intensity and their interpretation is ambiguous in literature.

Spectra presented in Fig. 7a have a characteristic broad peak at about 130 $\hbox {cm}^{-1}$, which is not due to selection rules for Raman scattering. Its presence and broadening is related to the defective structure of the $\delta$ phase. This cubic structure is related to the fluorite structure but has ordered defects in the oxygen sublattice in the 111 direction^25^. Another reason of this broadening may be due to the atomic disorder resulting from the random orientation of the lone-pair orbitals of Bi atoms. Additionally, thermodynamic metastability of the $\delta$-$\hbox {Bi}_2\hbox {O}_3$ phase at low temperature can lead to a phase transition or a partial decomposition of $\delta$-$\hbox {Bi}_2\hbox {O}_3$ into another phase^26^. The very wide peak at about 400 $\hbox {cm}^{-1}$ present in Fig. 7a–c may come from amorphous or glassy $\hbox {SiO}_2$ or $\hbox {SiO}_2$ with $\hbox {Al}_2\hbox {O}_3$ addition. The presence of Si and Al was confirmed for these samples in the SEM-EDS study. The Raman spectrum of vitreous silica is dominated by the region below 600 $\hbox {cm}^{-1}$. A major band occurs at 430 $\hbox {cm}^{-1}$. By adding $\hbox {Al}_2\hbox {O}_3$ to $\hbox {SiO}_2$, the intensity of this peak decreases, while the position practically does not change^27^. Various disordered $\hbox {Al}_2\hbox {O}_3$ phases quite often show broad peaks at ca 400 $\hbox {cm}^{-1}$, as well as 900 $\hbox {cm}^{-1}$. A weak peak at about 900 $\hbox {cm}^{-1}$ is observed in practically all samples under study (Fig. 7a–c). The high intensity of the peak around 400 $\hbox {cm}^{-1}$ (Fig. 7a) may indicate that the $\hbox {SiO}_2$ and $\hbox {Al}_2\hbox {O}_3$ phases are located on the surface of the $\hbox {Bi}_2\hbox {O}_3$ crystalline nuclei.

When comparing the Raman spectra (Fig. 7) of the same samples with different heat-treatment, one can conclude as follows. Initially, all the as-synthesized glassy samples showed a $\delta$ phase (peak at 620 $\hbox {cm}^{-1}$ and broad peak at 130 $\hbox {cm}^{-1}$) with addition of $\hbox {SiO}_2$ and/or $\hbox {Al}_2\hbox {O}_3$ (broad peak at 430 $\hbox {cm}^{-1}$) (Fig. 7a). Then, after heat-treatment at 630 °C, a slight ordering of the $\delta$ phase can be seen (samples from MQ-1100-30, MQ-1100-25, and MQ-1100-20 batches (Fig. 7b). Additionally, in the case of the MQ-1100-15 batch the presence of the $\gamma$ phase in the samples was observed (splitting of the peak at 130 $\hbox {cm}^{-1}$ and the characteristic peak at 538 $\hbox {cm}^{-1}$).

Thermal treatment at even higher temperatures resulted in more distinct transition to the $\gamma$ phase (batches MQ-1100-15, MQ-1100-20, Fig. 7c). In addition, the presence of the $\beta$ phase in the samples from these batches (peak at about 460 $\hbox {cm}^{-1}$) and even small amounts of the $\delta$ phase (peak at about 600–620 $\hbox {cm}^{-1}$) is noticeable. As for the MQ-1100-30, and MQ-1100-25 batches, the dominance of the $\delta$ phase is visible. These conclusions largely coincide with the results obtained in X-ray studies (Table 3).

It was shown that the pristine glasses contain noticeable amounts of Al and Si dopants. Therefore, it was natural to think of these glasses as ternary $\hbox {Bi}_2\hbox {O}_3$–$\hbox {Al}_2\hbox {O}_3$–$\hbox {SiO}_2$ system. In terms of optical results, this approach meets some difficulties. The values of bismuth oxide optical gap reported in the literature vary strongly, usually from 2.0 to 3.96 eV^28^. The exact value depends not only on the phase of $\hbox {Bi}_2\hbox {O}_3$, but also on the preparation route and the microstructure of the material (e.g. a thin film or polycrystalline ceramics)^29^. The value mostly reported for bulk samples is 2.75–2.8 eV^30^. Aluminum oxide and silicon oxide, regardless their exact structure, are well-known insulators. The reported values of the band gap for $\hbox {SiO}_2$, both crystalline (e.g. $\alpha$-quartz) and amorphous, are around 9–10 eV^31,32^. Bigger diversity is observed for aluminum oxide. For example, metastable $\theta$-$\hbox {Al}_2\hbox {O}_3$ exhibits an indirect ban dgap of 4.64 eV^33^, the stable $\alpha$-$\hbox {Al}_2\hbox {O}_3$ exhibits a band gap of 7.6 eV^34^ and amorphous alumina 7.0 eV^34^. A gradual shift of the curves and hence, estimated absorption edge energies, was observed in Ref.^35^ for binary $\hbox {Bi}_2\hbox {O}_3$–$\hbox {B}_2\,\hbox {O}_3$ glasses, where samples with higher concentration of boron oxide exhibited absorption edge in higher energies. In case of this study, there was no monotonic dependence of the optical band gap on the composition of the glasses. The values of the calculated optical band gap ranged from 2.42 to 2.73 eV, depending on the sample and the method of estimation. This is either because the Al and Si dopants did not affected significantly the mobility gap or the changes in concentrations were too small in this study to be reflected in the absorption measurements.

The Al and Si additives have a significant role in the whole process. Usually, $\delta$-like phase of the fluorite structure is stabilized to lower temperature with expensive dopants of rare earth elements, e.g., Y^36^, Yb^37^, Dy and Tm^38^, Th^39^. Si and Al dopants were reported to stabilize $\beta$ or $\gamma$ phases only in polycrystalline forms^40,41^. There is no evidence in the literature that these dopants may stabilize the $\delta$ phase by solid solution. Our studies have shown that the samples exhibited significantly different microstructure after crystallization. $\delta$ phase always existed in the form of small nanocrystallites embedded in a glassy matrix. Grains of the $\beta$ phase were always larger and the $\gamma$ phase appeared in micrometer-sized grains. It is an interesting observation that the position of the maximum in XRD patterns of glasses is similar to the (111) reflex of the $\delta$ phase or the (201) line of the $\beta$ phase (approx. $2\Theta = 28 ^{\circ }$), rather than alpha ($2\Theta = 27.4^{\circ }$). It may suggest that the short-range order of glass resembles these two unstable phases rather that the stable monoclinic alpha. However, to verify this statement, other experiments will be scheduled, i.e. nuclear magnetic resonance (NMR) measurements and calculation of pair distribution function from a wide range XRD patterns collected with a silver lamp.

To conclude, in this work we have shown that Al and Si dopants tend to diffuse from porcelain crucibles to a $\hbox {Bi}_2\hbox {O}_3$ batch during its melting at 1100 $^{\circ }$C. The concentration of Al was approximately the same, while the content of Si increased linearly with the increasing melting time (from 15 to 30 min). For even lower melting times, it was not possible to obtain a bismuthate glass using standard melt-quenching technique. In glasses heated to 630 $^{\circ }$C, the nanocrystallites of the $\delta$ phase appeared, which remained stable after cooling down to room temperature. Similarly, the heating up to 730 $^{\circ }$C resulted in appearance of a stable $\beta$ phase. However, in samples with lower Si content, additional micrometric crystallites of the $\gamma$ phase had a significant share.

While the exact role and position of Al and Si dopants in the structure has not been directly determined yet, our opinion is that the size and strain effects play the most dominant role in stabilization phenomenon. While Al/Si dopants facilitate the glass forming of bismuth oxide, the final stabilization is likely due to the confinement effects of nanocrystallites in the residual glassy matrix. The “power” of the size effect has already been proven by Dreyer et al.^42^, who synthesized stable free $\delta$-$\hbox {Bi}_2\hbox {O}_3$ nanoparticles with minute dopant concentrations. Confinement effects in a glassy matrix (i.e. involving the local stress) could have an even stronger effect on $\delta$-$\hbox {Bi}_2\hbox {O}_3$ stabilization, where no dopants would be necessary. Further experiments that could provide solid evidence for this phenomenon will be performed in the near future.

## Methods

Commercial polycrystalline $\hbox {Bi}_2\hbox {O}_3$ (monoclinic $\alpha$ phase) powder (Arcos Organics, 99.9% extra pure) was put into porcelain VWR crucibles and subsequently inserted into a chamber furnace (manufacturer: Czylok, Poland) preheated to 1100$^{\circ }$ C. The samples were kept in the furnace for 15–30 min (according to the description in Table 1) and then poured onto a stainless steel plate kept at room temperature and rapidly quenched with another plate. As a result, bulk transparent samples with orange tint and thickness ca 1 mm were obtained. As for reference, the same reagent was melted in a platinum crucible at 1100 $^{\circ }$C for 30 min and cooled down accordingly.

Thermal analyses were performed using TA SDT Q600 apparatus from room temperature to 775 $^{\circ }$C with the heating rate 10 $^{\circ }$C/min. Ca. 10 mg of powdered glasses were put in alumina crucibles. The experiments were carried out in argon flow (100 ml/min).

Glassy samples were subject to further thermal treatment. Pieces of the glassy materials were put into 8 porcelain crucibles. First part was put into a chamber furnace LIFT 3 (manufacturer: Neoterm, Poland) and heated up to 630 $^{\circ }$C, then kept 60 min at that temperature and cooled down to RT. The heating rate in this process was set to 1 $^{\circ }$C/min, and the cooling rate was set to 3 $^{\circ }$C/min. The procedure was performed in the air.

XRD studies of glasses and nanomaterials were carried out at room temperature in the air. A Malvern Panalytical Empyrean device was used with $K_{Cu\alpha }$ radiation and a nickel filter. The high-quality patterns were collected at a wide $2\Theta$ range from 10° to 115° using PIXcel3D detector. The phase composition was analyzed using HighScore Plus software and PDF4 2021 database.

The surface topography investigations were performed by using Scanning Electron Microscope, Hitachi SU8000 (made in Naka, Japan) at the accelerating voltage of 5 kV. The low voltage was applied to reduce charging effect on uncoated samples. The observations were done by using SE and BSE electrons. At the same accelerating voltage the chemical composition analysis was performed by using EDS made by Thermo Fisher. The microstructure of the samples was also investigated with scanning electron microscopy (SEM) using Zeiss Ultra plus apparatus in the Institute of High Pressure Physics, Polish Academy of Sciences. For this experiment, a thin layer of carbon was sputtered on the surface of the samples.

The Raman spectra were measured with an inVia Renishaw spectrometer, utilizing either the 514 nm of an Ar laser or the 633 nm line of a He–Ne laser as sample excitation. Each Raman spectrum was collected for 30 s (3 accumulations) at room temperature. Low laser beam powers were used to avoid the processes of structural degradation of the studied samples.

The absorption measurements were performed at room temperature using a Bentham PVE300 device equipped with a tunable light source (composed of xenon and halogen lamps), a monochromator, an integrating sphere and a silicon detector. Sample was placed between the integrating sphere and the light source. The light was directed at the sample using a set of mirrors. The procedure consisted of measuring the light intensity after it passes through the sample and dividing it by the incident light intensity. Therefore, the output value from this measurement was the transmission of the sample. Transmission is calculated into the absorption coefficient $\alpha$ using the following formulas: $T=\frac{I}{I_0}$, $\alpha = \frac{1}{d} \ln \left( \frac{1}{T} \right) = \frac{1}{d} \ln \left( \frac{I_0}{I} \right)$, where: *T *— transmission, *I *— light intensity after it passes through the sample, $I_0$ — incident light intensity, *d* — thickness of the sample.
